# Supplementary material for: Large Paraumbilical Vein Shunts Increase the Risk of Overt Hepatic Encephalopathy after Transjugular Intrahepatic Portosystemic Shunt Placement
Source: J Clin Med. 2022 Dec 25;12(1):158. doi: 10.3390/jcm12010158 (PMC9821527; doi:10.3390/jcm12010158)
Supplement: Supplementary file 1 [file jcm-12-00158-s001.zip › Table S1.pdf]

Table S1. Baseline Characteristics of Patients before PSM.

Parameter	L-PUV (n=27)	Control group (n=191)	P Value
Sex (male), n (%)	15 (55.6%)	108 (56.5%)	0.923
Age (years)	54.0 (42.0-66.0)	59.0 (49.0-68.0)	0.102
Viral hepatitis	15 (55.6%)	97 (50.8%)	0.642
Ascites (present), n (%)	13 (48.1%)	104 (54.5%)	0.539
Previous diabetes, n (%)	4 (14.8%)	36 (18.8%)	0.604
Previous splenectomy, n (%)	0	31 (16.2%)	0.003
Presence of gastric varices, n (%)	5 (18.5%)	10 (5.2%)	0.027
Portal vein thrombosis, n (%)	7 (25.9%)	55 (28.8%)	0.755
Total bilirubin (umol/L)	24.1 (16.0-39.5)	21.9 (15.2-28.9)	0.176
Albumin (g/L)	28.6 (26.7-33.6)	30.7 (27.3-34.7)	0.319
Creatinine (umol/L)	59.4 (45.9-67.2)	62.2 (53.0-72.9)	0.071
Prothrombin time (S)	16.2 (14.7-18.7)	15.5 (14.1-17.0)	0.074
International normalised ratio	1.38 (1.23-1.60)	1.31 (1.20-1.46)	0.126
Platelet ($\times 10^9$/L)	59.0 (40.0-73.0)	69.0 (49.0-99.0)	0.040
White blood cell ($\times 10^9$/L)	3.3(2.0-7.3)	5.0 (2.7-8.5)	0.036
Child-Pugh score (points)	7.0 (6.0-8.0)	7.0 (6.0-8.0)	0.976
Child-Pugh grade, n (%)			0.399
A	8 (29.6%)	69 (36.1%)	
B	15 (55.6%)	103 (53.9%)	
C	4 (14.8%)	19 (9.9%)	
MELD score (points)	12.0 (10.0-13.0)	11.0 (9.0-13.0)	0.102
Diameter of LPV	13.2 (10.7-14.8)	10.9 (9.2-12.5)	0.001
Diameter of RPV	11.6 (8.1-13.0)	10.7 (8.7-12.0)	0.439
Diameter of MPV	16.9 (13.8-20.6)	15.6 (13.9-17.8)	0.258
Esophageal variceal grade			0.285
0	2 (7.4%)	2 (1.1%)	
I	1 (3.7%)	2 (1.1%)	
II	2 (7.4%)	19 (10.0%)	
III	22 (81.5%)	167 (87.9%)	
Portal vein puncture			0.316
LPV	19 (70.4%)	123 (64.4%)	
RPV	7 (25.9%)	33 (17.3%)	
MPV	1 (3.7%)	35 (18.3%)	

Pre-TIPS PPG (mmHg)	21.0 (18.0-21.0)	21.0 (17.0-25.0)	0.109
Post-TIPS PPG (mmHg)	7.0 (6.0-9.8)	8.0 (6.0-11.0)	0.261
Diameter of expansion balloon catheters, n (%)			0.731
6mm	6 (22.2%)	37 (19.4%)	
8mm	21 (77.8%)	154 (80.6%)	
Duration of follow-up (months)	22.0 (12.0-43.0)	33.0 (20.0-47.0)	0.061

Note: Data are expressed as median (IQR) or n (%). Abbreviations: PUV, paraumbilical veins; MELD, Model for End-Stage Liver Disease; LPV, left portal vein; RPV, right portal vein; MPV, main portal vein; EV, esophageal varices; TIPS, transjugular intrahepatic portosystemic shunt; PPG, portal pressure gradient.
