# Supplementary material for: Large Paraumbilical Vein Shunts Increase the Risk of Overt Hepatic Encephalopathy after Transjugular Intrahepatic Portosystemic Shunt Placement
Source: J Clin Med. 2022 Dec 25;12(1):158. doi: 10.3390/jcm12010158 (PMC9821527; doi:10.3390/jcm12010158)
Supplement: Supplementary file 1 [file jcm-12-00158-s001.zip › Table S2.pdf]

Table S2. Characteristics of PUV.

**Parameter**	
**L-PUV**	**27/218 (12.4%)**
diameter of PUV (mm)	9.8 (8.8-14.0)
cross-sectional PUV area (mm^2)	75.4 (55.4-158.4)
PUV diameter ≥ 8mm	23/27 (85.2%)
PUV/MPV ratio >0.5, n (%)	25/27 (92.6%)
cross-sectional PUV area >83 mm^2, n (%)	13/27 (48.1%)

Abbreviations: PUV, paraumbilical veins; MPV, main portal vein; L-PUV, large paraumbilical veins.
