# Supplementary figures and images for: Large Paraumbilical Vein Shunts Increase the Risk of Overt Hepatic Encephalopathy after Transjugular Intrahepatic Portosystemic Shunt Placement
Source: J Clin Med. 2022 Dec 25;12(1):158. doi: 10.3390/jcm12010158 (PMC9821527; doi:10.3390/jcm12010158)

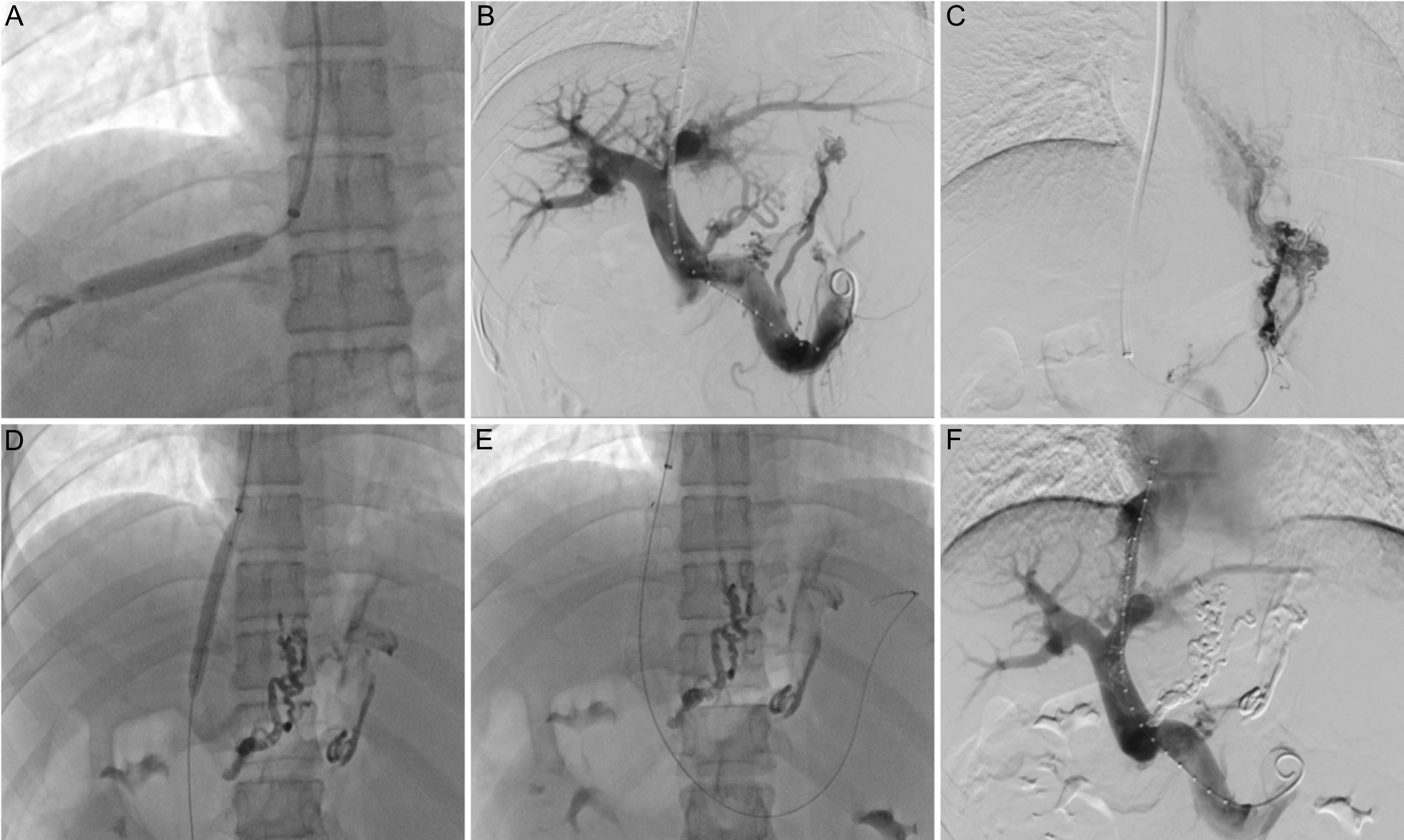

Supplement: Supplementary file 1 [file jcm-12-00158-s001.zip › Figure S1.jpg]

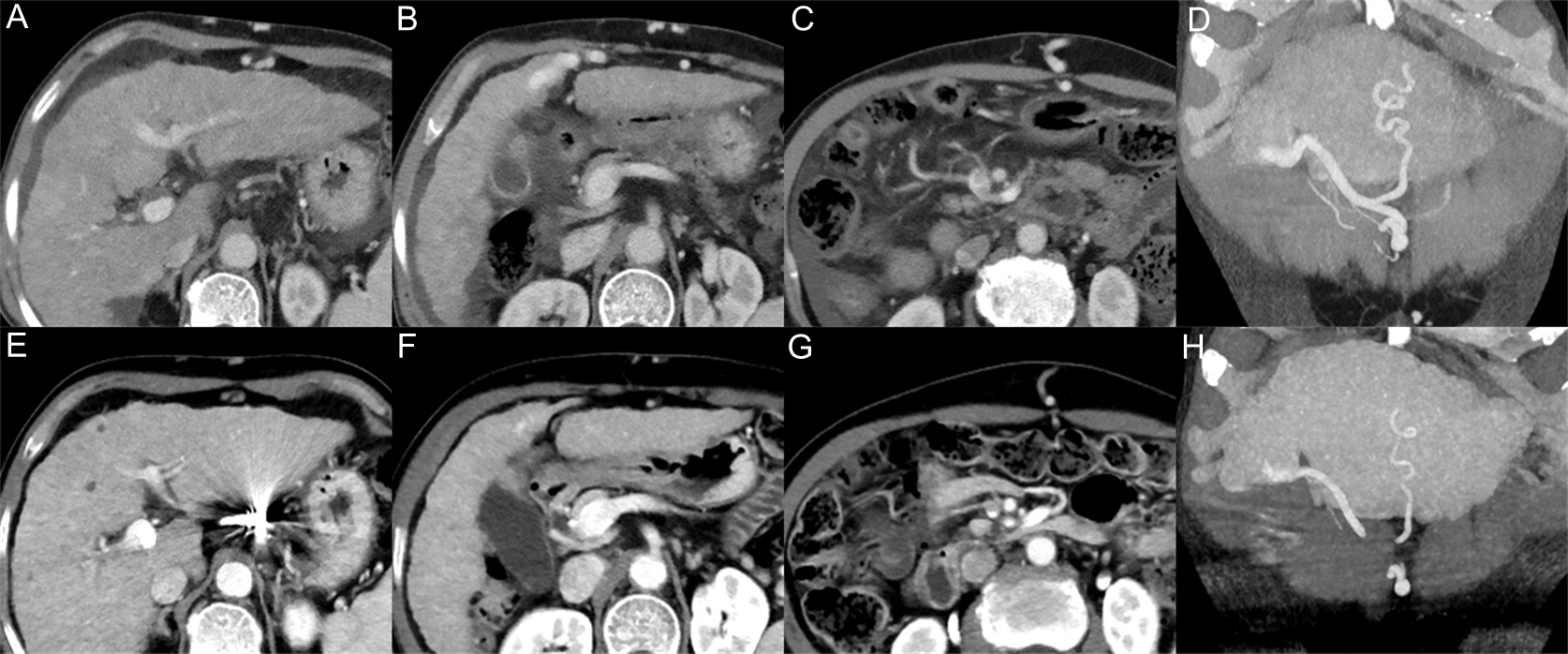

Supplement: Supplementary file 1 [file jcm-12-00158-s001.zip › Figure S2.jpg]

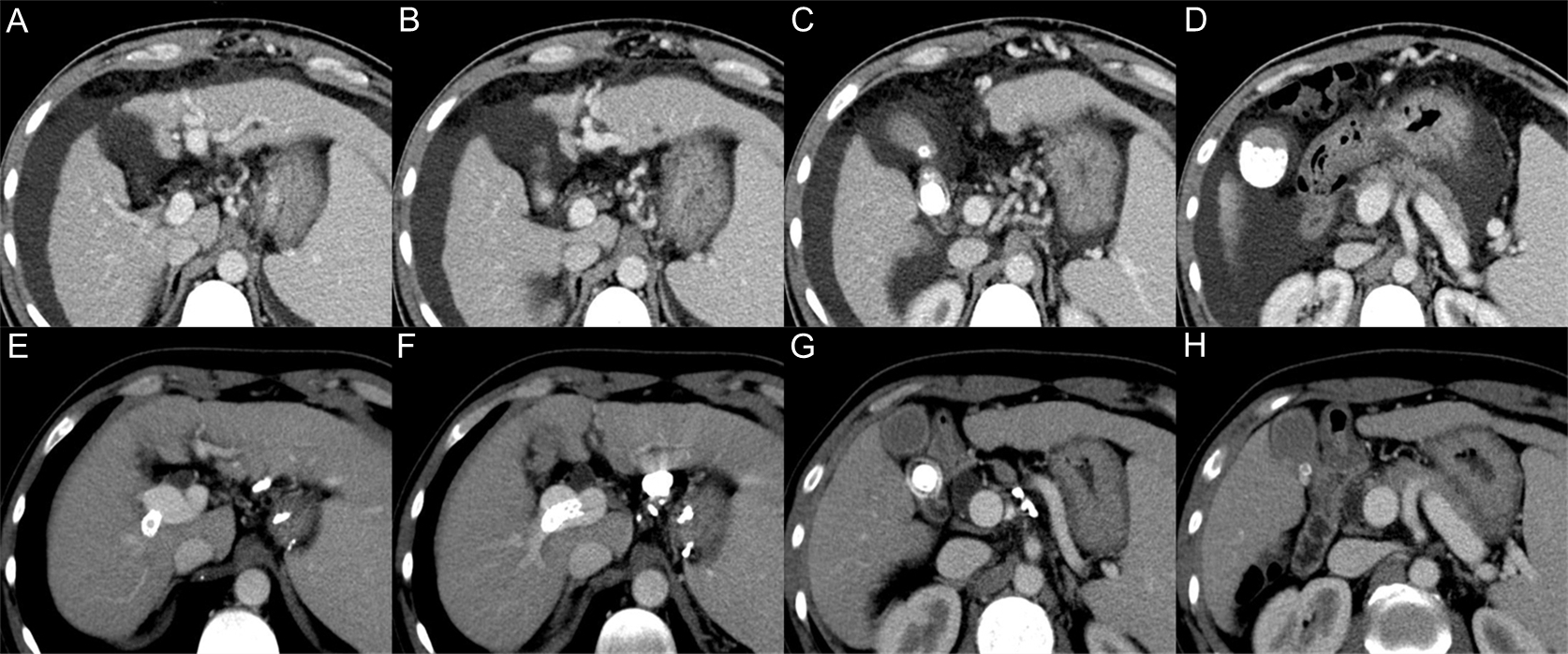

Supplement: Supplementary file 1 [file jcm-12-00158-s001.zip › Figure S3.jpg]
